# Post VV-ECMO Weaning Hyperinflammation—Can Prophylactic Hemoadsorption Treatment Prevent Complications?

**DOI:** 10.3390/medicina59101818

**Published:** 2023-10-12

**Authors:** Pedja Kovacevic, Sasa Dragic, Milka Jandric, Danica Momcicevic, Sandra Topolovac, Vedrana Malesevic, Tijana Kovacevic, Marijana Matejic-Spasic, Tanja Knezevic, Biljana Zlojutro

**Affiliations:** 1 Medical Intensive Care Unit, University Clinical Centre of the Republic of Srpska, 78000 Banja Luka, Bosnia and Herzegovina; drdragics@gmail.com (S.D.); milka.jandric@kc-bl.com (M.J.); danica.momcicevic@kc-bl.com (D.M.); kovacevic.sandra@yahoo.com (S.T.); vedrana.malesevic@kc-bl.com (V.M.); tijanamar@gmail.com (T.K.); biljana.zlojutro@kc-bl.com (B.Z.); 2 Faculty of Medicine, University of Banja Luka, 78000 Banja Luka, Bosnia and Herzegovina; 3 CytoSorbents Europe GmbH, 12587 Berlin, Germany; marijana.matejic@cytosorbents.com

**Keywords:** ECMO, hyperinflammation, hemoadsorption, CytoSorb

## Abstract

Acute respiratory distress syndrome (ARDS) is a severe clinical condition characterized by acute respiratory failure and a high mortality risk despite conventional mechanical ventilatory support. Veno-venous extracorporeal membrane oxygenation (vvECMO) has emerged as an effective life-support technology for patients with ARDS. However, complications may arise following the decannulation of vvECMO, with a relatively frequent development of systemic hyperinflammation (SHI). Among the various treatment strategies for SHI, the use of hemoadsorption with CytoSorb^®^ has shown promising results in removing excessive levels of cytokines and attenuating the hyperinflammatory response. In this case series, we present three critically ill patients with ARDS secondary to pneumonia who underwent vvECMO and subsequently received prophylactic hemoadsorption with CytoSorb^®^ following decannulation as a part of our clinical practice. This case series aims to describe the potential positive effects of hemoadsorption in preventing the development of SHI after vvECMO decannulation in ARDS patients.

## 1. Introduction

Veno-venous extracorporeal membrane oxygenation (vvECMO) is a technology used to support patients with acute respiratory distress syndrome (ARDS) and high mortality risk despite the use of conventional mechanical ventilatory support [1,2]. The primary purpose of ECMO is to increase oxygenation and maintain adequate gas exchange with or without hemodynamic support to allow time for the recovery of the respiratory system. Once the criteria for adequate tissue perfusion and respiratory system recovery are met, patients are ultimately decannulated from vvECMO [3]. Conversely, there are numerous complications that may occur after weaning from vvECMO, compromising the success of the treatment, sometimes even to the extent of being life threatening [4,5]. Complex and multi-faceted innate inflammatory response to the artificial materials of the extracorporeal circuit contribute to the development of systemic hyperinflammation (SHI) [4,5,6,7,8]. Systemic inflammation can also appear due to the nature of the underlying disease or secondary infection, prolonged mechanical ventilation, aspiration, or extensive vascular injury [4]. SHI has already been identified by researchers as a clinical entity that follows ECMO decannulation and is described in the literature. Worse, and sometimes lethal, outcomes have been observed in those patients who had evidence of infection and concomitant sepsis following decannulation [5,6,7,8,9]. This severe condition, associated with a dysregulated immune response (“cytokine storm”) to either an infectious or non-infectious trigger, is often deleterious, especially if accompanied by vasoplegic shock. In addition to the standard treatment of hyperinflammation, cytokine removal with extracorporeal blood purification techniques represents a very attractive adjuvant treatment option. Over the past decade, new adsorption materials were created and tested for use in patients with severe elevation of cytokines. CytoSorb^®^ (CytoSorbents Inc., Princeton, NJ, USA) is one of the hemoadsorbers approved in Europe, with a large effective surface area of approximately 40,000 m^2^ [10]. Literature data about the effect of CytoSorb^®^ therapy on the prevention of systemic hyperinflammation after vvECMO decannulation in ARDS patients are scarce.

This case series was compiled in order to describe the possible positive effects of the use of CytoSorb^®^ for preventing SHI following vvECMO decannulation in patients with ARDS. There were no published reports on such clinical practice thus far.

### 1.1. Case Series Report

**Figure 1:**
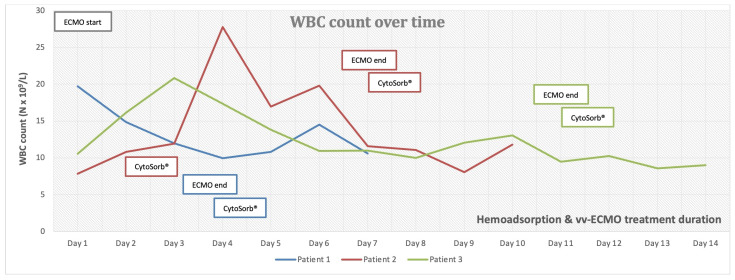
White blood cell (WBC) count over time.

**Figure 2:**
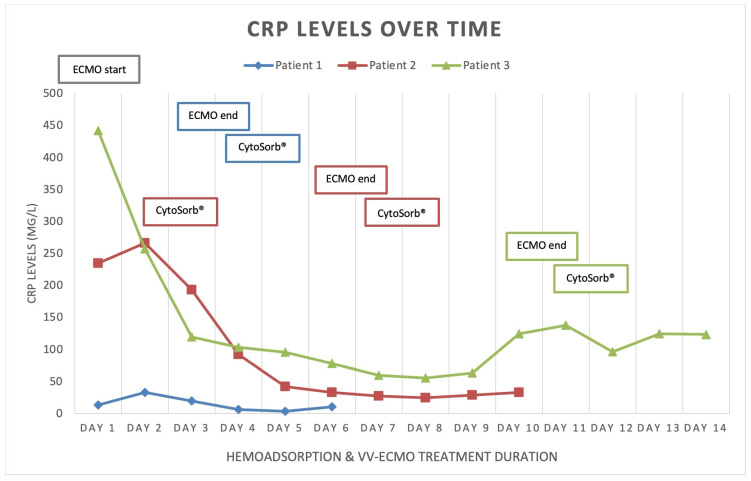
C-reactive protein (CRP) levels over time.

**Figure 3:**
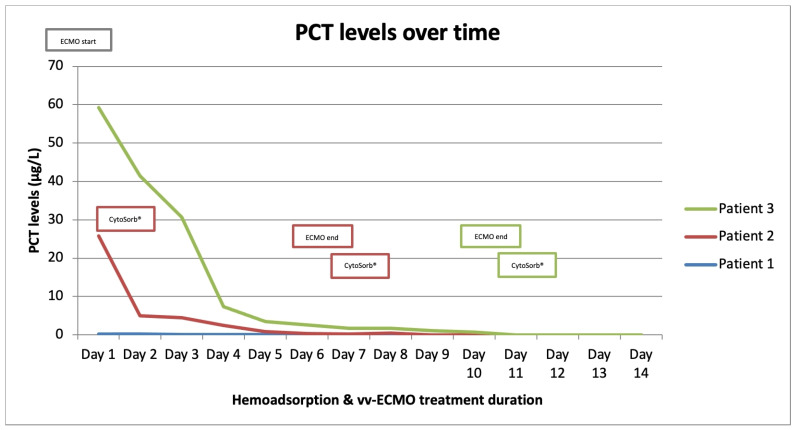
Procalcitonin (PCT) levels over time.

We present three critically ill patients with acute respiratory insufficiency caused by pneumonia (non-COVID-19) complicated by ARDS of different etiologies. These patients were admitted and treated in the medical intensive care unit (MICU) of the University Clinical Centre Republic of Srpska (UCC RS). Routinely obtained clinical and laboratory data were retrospectively collected and analyzed. Table 1 and Table 2 provide a structured overview of all three cases. The time-course of inflammatory markers is presented graphically in Figure 1, Figure 2 and Figure 3, which, together with the clinical course of the disease, served to assess the outcomes of the three presented cases. There were no device-related adverse events and none of the three patients expressed any intolerability to hemadsorption treatment.

The data were collected in accordance with the Declaration of Helsinki. The ethical review and approval were waived due to the retrospective nature of this report, which utilizes routinely obtained de-identified clinical and laboratory data. Written informed consent was obtained from the patients for publication of their respective case reports.

### 1.2. Patient 1

Patient 1 was a 33-year-old female, with twin pregnancy at week 23 of gestation admitted to the MICU for deterioration of acute respiratory failure. The patient had been previously treated on the gynecology ward for nineteen days, where she underwent cervical cerclage placement due to the risk of premature birth. Seven days before MICU admission, the patient developed fever, cough, headache, and fatigue. Due to the rapid worsening of her symptoms and increased oxygen demand, the patient was transferred to the MICU of UCC RS. At the time of admission, she was conscious but extremely fatigued, tachypneic and dyspneic, afebrile, but with oxygen saturation (SpO_2_) of 92% (non-rebreather (NRB) mask, flow 15 L/min), respiratory rate (RR) of 30 breaths/min, heart rate (HR) 80 beats/min, and blood pressure (BP) of 105/75 mmHg (Table 1). An increase in inflammatory parameters was observed (Table 2, Figure 1, Figure 2 and Figure 3, blue), and the arterial blood gas analysis (ABG) indicated metabolic acidosis with electrolyte disturbance. Immediately after admission, the patient was treated with noninvasive ventilation (NIV), but within two hours, this had proved insufficient; therefore, endotracheal intubation was performed. Due to the severe ARDS, the patient was ventilated in a prone position. During the course of the same day, her respiratory failure worsened despite optimal mechanical ventilatory support, indicating the need for the initiation of vvECMO therapy. Over the following few days, her clinical condition improved significantly, and therefore she was successfully weaned from vvECMO on day 4. As per our internal protocol, immediately after decannulation, the patient was connected to the extracorporeal circuit for prophylactic 24 h hemoadsorption treatment with one CytoSorb^®^ adsorber. No signs of systemic hyperinflammation were noted over the following days (Table 2, Figure 1, Figure 2 and Figure 3, blue). Her clinical condition gradually improved, and on MICU day 10, she was successfully extubated. After another 4 days, she was eventually transferred back to the gynecology ward for further treatment. Fifty days after transfer, she delivered two healthy babies.

### 1.3. Patient 2

Patient 2 was a 35-year-old man who was admitted to the MICU of UCC RS due to deteriorating acute respiratory failure with the development of septic shock. He was treated for one day in the regional hospital for bilateral pneumonia. The disease had started six days before MICU admission with fever, fatigue, dyspnea, and cough with hemoptysis. Previously, he was healthy and without any comorbidities. At the time of admission, he was conscious but lethargic, pale, and damp with cold sweat. The patient had a high fever (39 °C), was tachypneic and dyspneic, with a low SpO2 of 86% (NRB mask, flow 15 L/min), high RR of 33 breaths/min, high HR of 130 beats/min, and his BP was 130/80 mmHg. Laboratory findings revealed signs of acute kidney injury, low values of hemoglobin and platelets, high values of aminotransferases and bilirubin, as well as extremely increased inflammatory markers (Table 1 and Table 2, Figure 1, Figure 2 and Figure 3, red). Chest imaging with computed tomography (CT) scan depicted multifocal pneumonic bilateral infiltrates affecting 80% of the right lung and 50% of the left lung, but no pulmonary embolism was seen. Initially, the patient was treated with NIV. After a few hours, NIV failed to provide sufficient oxygen delivery, and therefore endotracheal intubation and ventilation were performed due to the rapid worsening of his acute respiratory failure and development of severe ARDS. On MICU day 2, the patient’s condition deteriorated, and the repeated CT scan revealed disease progression. The patient was put in the prone position and inhaled nitric oxide (iNO) was administered as a rescue therapy for the persistent hypoxemia. Unfortunately, due to refractory respiratory failure despite all measures, vvECMO had to be initiated. During vvECMO treatment and the “lung rest” mode of mechanical ventilation, the patient was repeatedly prone positioned with the aim of improving ventilation–perfusion mismatch. On MICU day 7, the patient’s condition again deteriorated after the onset of septic shock. All necessary resuscitation measures (sepsis bundle) were employed and, since the criteria for the initiation of renal replacement therapy were met, the continuous method of veno-venous hemodiafiltration (CVVHDF) with hemoadsorber (CytoSorb^®^) was commenced as an adjunctive therapy for another 24 h. The antibiotic therapeutic scheme was corrected according to the microbiological and laboratory findings, and the antibiotic dosages were adjusted according to the dosing guidance for CVVHDF. Over the following days, the patient’s condition began to improve, and on vvECMO day 7 (MICU day 9), he was decannulated. Following decannulation, the patient received another hemoadsorption treatment with one cartridge of CytoSorb^®^ for SHI prophylaxis for a minimum duration of 12 h. No signs of systemic hyperinflammation were noted over the following days (Table 2, Figure 1, Figure 2 and Figure 3, red); however, the C-reactive protein (CRP) levels failed to drop below physiological threshold over the whole course of treatment. On MICU day 21, percutaneous dilatational tracheostomy was performed. The patient’s clinical condition continued to improve, and weaning from mechanical ventilation was thus initiated. After a total of 34 days in the MICU, the patient completely recovered, and was transferred to the pulmonary ward. Six days later, the patient was able to be discharged home.

### 1.4. Patient 3

Patient 3 was a 50-year-old woman who was admitted to MICU due to sepsis and respiratory failure caused by bilateral pneumonia. She reported a cough, sore throat, fever, and fatigue for eight days. Additionally, she had a history of hypertension. The diagnosis of bilateral pneumonia was confirmed through radiological imaging (chest X-ray). Upon admission to the MICU, she was conscious, alert, tachypneic, dyspneic, with a low SpO2 of 90% (NRB mask, flow 15 L/min), high RR of 33 breaths/min, high HR of 115 beats/minute, normal BP, and afebrile. Laboratory tests showed elevated markers of inflammation (Table 2). Initially, she was placed on NIV, but her respiratory dysfunction worsened; thus, she was intubated and connected to controlled mechanical ventilation. In order to improve oxygenation, iNO was added to her therapy and she was prone positioned while on mechanical ventilation, although this only temporarily stabilized her gas exchange. The next day, her respiratory insufficiency progressed further, and there was no response to the implemented measures; therefore, she was eventually connected to vvECMO. Over the period of 11 days on vv ECMO, the patient remained hemodynamically stable, with a slight improvement in respiratory function (shown in Figure 1, Figure 2 and Figure 3, green). On MICU day 12, after significant improvement, weaning from vvECMO was performed. Subsequently, a dialysis central venous catheter was placed for CRRT, which served as a platform for hemoadsorption with CytoSorb^®^. This procedure was initiated to prevent the possible development of SHI after vvECMO decannulation. After 24 h of single-hemoadsorber therapy, the prophylactic hemoadsorptive treatment was terminated. The white blood cell count and procalcitonin level were within the normal range 48 h post-decannulation, but the CRP remained highly elevated despite an observed generalized attenuation of inflammation (Table 2). To facilitate weaning from prolonged mechanical ventilation, a percutaneous dilatational tracheotomy was performed on MICU day 17. Furthermore, analgosedation was discontinued so that the patient was alert, remaining conscious, and breathing with the assistance of a ventilator, with no signs of an inflammatory response. The process of weaning off the ventilator was slow. On MICU day 32, tracheostomy decannulation was performed. In order to continue the recovery process, the patient was transferred to the rehabilitation center on day 44 of her stay in the MICU.

## 2. Discussion/Conclusions

The literature has extensively documented the clinical relationship between ECMO and systemic inflammation [8]. Introducing blood into an extracorporeal system is known to trigger a strong innate humoral and cellular response that was previously described in the literature as systemic inflammatory response syndrome (SIRS). Numerous downstream effects have been established, including dysregulation of leukocyte function, complement activation, and endothelial and thrombotic processes [4,7,8,11]. This interaction between coagulation, complement, and endothelial systems initiates and perpetuates a pro-inflammatory environment with cellular and cytokine involvement. If left uncontrolled, this can result in vascular and end-organ damage [11,12,13,14,15]. Furthermore, if this condition evolves into a dysregulated immune response like that seen in sepsis, with excessive both pro- and anti-inflammatory cytokine activity, the risk of death becomes overwhelming [16]. Several studies have shown that SHI commonly occurs after decannulation from vvECMO support, with an overall incidence of approximately 50%. This incidence remains similar in patients with or without concurrent COVID-19 infection [5,6,7,8]. Despite the older age and longer duration of vvECMO support in COVID-19 patients, these factors do not significantly affect the incidence of SHI or relevant outcomes. Initially, it was hypothesized that prolonged cannulation might lead to endothelial activation and sustained pro-inflammatory activity [5]. On the other hand, the presence of bacterial biofilm on the cannulas’ surfaces cannot be excluded, even in patients without proven bacteriemia [17,18]. The critical time for the development of inflammatory response is shown to be 48 h [19]. However, the risk factors for the development of the post-decannulation SHI phenomenon remained undetermined [20]. Consequently, the term “vvECMO gap” was introduced, represented by the patients that are successfully weaned off ECMO but still die during their hospital stay, most often from multiorgan failure and sepsis [21]. We have observed this gap in our institution as well, thus prompting us to seek potential solutions to minimize it. Adjunct hemoadsorption has increasingly been utilized to target underlying hyperinflammation derived from ARDS. A systematic review on the combined use of CytoSorb^®^ together with vvECMO found significant improvements in clinical outcomes associated with hemoadsorptive treatment—better PaO_2_/FiO_2_ ratio, reduced levels of CRP and interleukin-6, as well as favorable trends in norepinephrine dosages and survival [22]. Similarly, the potential mortality benefit was also observed in COVID-19 patients who required vvECMO for ARDS [23]. To the best of our knowledge, however, prophylactic use of hemoadsorption after decannulation has not been described in the literature so far, making our case series the first one in this field of application. This series demonstrates that vvECMO and concomitant extracorporeal blood purification with cytokine adsorber can potentially be a life-saving intervention when conventional methods fail to treat severe pneumonia and sepsis. The treatment approach involved a multidisciplinary strategy. Firstly, broad-spectrum antibiotic therapy was used for infection control, followed by the utilization of vvECMO for respiratory support and then attenuation of the hyperinflammatory response using the CytoSorb^®^ hemoadsorber. In all three cases, the hemoadsorber was also deployed immediately after weaning to prevent possible post-decannulation SHI. No patient required any vasopressor support throughout the course of the vvECMO treatment and after. Although cytokine values before and after decannulation and separation from vvECMO were not available, the initial inflammatory parameters and parameters 48 h after the use of the hemoadsorber in most parts demonstrated the absence of systemic hyperinflammation, both clinically and biochemically (Table 2, Figure 1, Figure 2 and Figure 3). The observed reduction in inflammatory markers correlated with improved clinical findings, as well as the respiratory and hemodynamic status of the patients in this case series. Although a causal relationship could not be established, we believe that the use of CytoSorb^®^ to control the (anticipated) excessive inflammatory response contributed to the patients’ stabilization and prevented the occurrence of post-decannulation SHI. Additional research is needed to better understand the impact of hemoadsorption therapies such as CytoSorb^®^ on the prevention of hyperinflammation after weaning from vvECMO, and its potential role in vvECMO gap reduction.

## Figures and Tables

**Table 1:** Demographic and clinical characteristics of the observed patients on MICU admission, and the overall vvECMO treatment duration.

	Patient 1	Patient 2	Patient 3
**Sex**	♀	♂	♀
**Age (years)**	33	35	50
**BMI**	21	26.1	24.5
**Comorbidity**	/	/	HTA
**HR/min**	80	130	115
**RR/min**	30	33	33
**MAP (mmHg)**	80	85	80
**FiO_2_ (%)**	1.0	1.0	1.0
**PEEP (cm H_2_O)**	16	22	16
**PaO_2_/FiO_2_ (ratio)**	34.4	84	74
**Murray Score (Lung Injury Score)**	3.8	3.5	4
**Vasopressors**	/	/	/
**Microbial isolates** (BAL—bronchoalveolar lavage; HC—hemoculture)	BAL: *Klebsiella* spp. 10^5^	BAL: Acinetobacter	BAL: Acinetobacter HC: Staphylococcus epidermidis
**Days on vvECMO**	4	7	11
*Different colors in columns correspond to colors of graphs and other elements in Figure 1, Figure 2 and Figure 3 belonging to appropriate patients.*			

**Table 2:** Longitudinal presentation of laboratory findings (hematological, biochemical, and inflammatory) of observed patients.

Laboratory Findings	Patient N^o^ 1			Patient N^o^ 2			Patient N^o^ 3		
	vvECMO Day 1	Decannulation Day	48h after Decannulation	vvECMO Day 1	Decannulation Day	48h after Decannulation	vvECMO Day 1	Decannulation Day	48h after Decannulation
**Leucocytes** (N × 10^9^/L)	19.7	13.6	10.5	2.7	3.2	3.9	10.5	9.5	8.6
**Platelets** (N × 10^9^/L)	202.0	196.0	169.0	30.0	94.0	104.0	283.0	95.0	108.0
**Creatinine** (µmol/L)	52.0	47.0	45.0	506.0	131.0	211.0	81.0	117.0	72.0
**AST** (U/L)	151.0	122.0	133.0	112.0	33.0	69.0	95.0	180.0	108.0
**ALT** (U/L)	288.0	269.0	286.0	59.0	81.0	211.0	286.0	211.0	86.0
**Bilirubin total** (µmol/L)	44.8	33.6	50.0	72.0	31.0	28.7	12.3	34.6	31.5
**Bilirubin direct** (µmol/L)	24.7	22.7	31.9	53.4	14.3	20.1	8.6	24.8	21.5
**CRP** (mg/L)	13.1	3.7	10.8	234.8	24.9	32.9	442	137.7	124.1
**Procalcitonin** (µg/L)	0.2	0.07	0.06	25.6	0.3	0.2	27.5	1.3	0.7
**Lactate** (mmol/L)	1.3	0.8	1.0	1.4	1.6	1.2	4.2	1.8	0.8
**Temperature** (°C)	37.1	36.3	36.6	36.6	36.5	36.8	36.5	36.7	36.7
*Different colors in columns correspond to colors of graphs and other elements in Figure 1, Figure 2 and Figure 3 belonging to appropriate patients.*									

## Data Availability

Data will be made available upon request within a reasonable timeframe.

## References

[1] Abrams D., Ferguson N.D., Brochard L., Fan E., Mercat A., Combes A., Pellegrino V., Schmidt M., Slutsky A.S., Brodie D. (2019). ECMO for ARDS: From salvage to standard of care?. Lancet Respir. Med..

[2] Tisminetzky M., Ferreyro B.L., Fan E. (2022). Extracorporeal Membrane Oxygenation in COVID-19. Crit. Care Clin..

[3] Wieruszewski P.M., Ortoleva J.P., Cormican D.S., Seelhammer T.G. (2023). Extracorporeal Membrane Oxygenation in Acute Respiratory Failure. Pulm. Ther..

[4] Chakraborty A., Majumdar H.S., Das W., Chatterjee D., Sarkar K. (2023). Discontinuation of ECMO-a review with a note on Indian scenario. Indian J. Thorac. Cardiovasc. Surg..

[5] Kirupaharan P., Blazoski C., Hilton R., Feduska E., Leong R., Baram M. (2023). Systemic Inflammatory Response Syndrome After Extracorporeal Membrane Oxygenation Decannulation in COVID-19 Patients. Cureus.

[6] Guan W.J., Ni Z.Y., Hu Y., Liang W.H., Ou C.Q., He J.X., Liu L., Shan H., Lei C.-L., Hui D.S.C. (2020). Clinical Characteristics of Coronavirus Disease 2019 in China. N. Engl. J. Med..

[7] Ki K.K., Passmore M.R., Chan C.H.H., Malfertheiner M.V., Bouquet M., Cho H.J., Suen J.Y., Fraser J.F. (2019). Effect of ex vivo extracorporeal membrane oxygenation flow dynamics on immune response. Perfusion.

[8] Warren O.J., Watret A.L., de Wit K.L., Alexiou C., Vincent C., Darzi A.W., Athanasiou T. (2009). The inflammatory response to cardiopulmonary bypass: Part 2--anti-inflammatory therapeutic strategies. J. Cardiothorac. Vasc. Anesth..

[9] Shekar K., Badulak J., Peek G., Boeken U., Dalton H.J., Arora L., Bishoy Z., Kollengode R., Joanne S., Bindu A. (2020). Extracorporeal Life Support Organization Coronavirus Disease 2019 Interim Guidelines: A Consensus Document from an International Group of Interdisciplinary Extracorporeal Membrane Oxygenation Providers. ASAIO J..

[10] Persic V., Jerman A., Malgaj Vrecko M., Berden J., Gorjup V., Stecher A., Lukic M., Jereb M., Taleska Stupica G., Gubensek J. (2022). Effect of CytoSorb Coupled with Hemodialysis on Interleukin-6 and Hemodynamic Parameters in Patients with Systemic Inflammatory Response Syndrome: A Retrospective Cohort Study. J. Clin. Med..

[11] Vallhonrat H., Swinford R.D., Ingelfinger J.R., Williams W.W., Ryan D.P., Tolkoff-Rubin N., Benedict C.A., Manljel P. (1999). Rapid activation of the alternative pathway of complement by extracorporeal membrane oxygenation. ASAIO J..

[12] Boyle E.M., Jr Pohlman T.H., Johnson M.C., Verrier E.D. (1997). Endothelial cell injury in cardiovascular surgery: The systemic inflammatory response. Ann. Thorac. Surg..

[13] Haneke F., Schildhauer T.A., Schlebes A.D., Strauch J.T., Swol J. (2016). Infections and Extracorporeal Membrane Oxygenation: Incidence, Therapy, and Outcome. ASAIO J..

[14] Millar J.E., Fanning J.P., McDonald C.I., McAuley D.F., Fraser J.F. (2016). The inflammatory response to extracorporeal membrane oxygenation (ECMO): A review of the pathophysiology. Crit. Care.

[15] Edmunds L.H. (1998). Inflammatory response to cardiopulmonary bypass. Ann. Thorac. Surg..

[16] Datzmann T., Traeger K. (2018). Extracorporeal membrane oxygenation and cytokine adsorption. J. Thorac. Dis..

[17] Yeo H.J., Yoon S.H., Lee S.E., Cho W.H., Kim D., Jeon D., Kyung-Hwa S., Seong K.Y. (2018). Bacterial Biofilms on Extracorporeal Membrane Oxygenation Catheters. ASAIO J..

[18] Yu Y., Kim Y.H., Cho W.H., Son B.S., Yeo H.J. (2021). Biofilm microbiome in extracorporeal membrane oxygenator catheters. PLoS ONE.

[19] Esposito E.C., Jones K.M., Galvagno S.M., Kaczorowski D.J., Mazzeffi M.A., DiChiacchio L., Deatrick K.B., Madathil R.J., Herrold J.A., Rabinowitz R.P. (2021). Incidence of healthcare-associated infections in patients with fever during the first 48 hours after decannulation from veno-venous extracorporeal membrane oxygenation. Perfusion.

[20] Thangappan K., Cavarocchi N.C., Baram M., Thoma B., Hirose H. (2016). Systemic inflammatory response syndrome (SIRS) after extracorporeal membrane oxygenation (ECMO): Incidence, risks and survivals. Heart Lung.

[21] Heuts S., Makhoul M., Mansouri A.N., Taccone F.S., Obeid A., Belliato M., Broman L.M., Broman L.M., Meani P., Raffa G.M. (2022). Defining and understanding the “extra-corporeal membrane oxygenation gap” in the veno-venous configuration: Timing and causes of death. Artif. Organs.

[22] Akil A., Napp L.C., Rao C., Klaus T., Scheier J., Pappalardo F. (2022). Use of CytoSorb(c) Hemoadsorption in Patients on Veno-Venous ECMO Support for Severe Acute Respiratory Distress Syndrome: A Systematic Review. J. Clin. Med..

[23] Hayanga J.W.A., Song T., Durham L., Garrison L., Smith D., Molnar Z., Scheier J., Deliargyris E.N., Moazami N. (2023). Extracorporeal hemoadsorption in critically ill COVID-19 patients on VV ECMO: The CytoSorb therapy in COVID-19 (CTC) registry. Crit. Care.

